# Effects of Natural and Anthropogenic Change on Habitat Use and Movement of Endangered Salt Marsh Harvest Mice

**DOI:** 10.1371/journal.pone.0108739

**Published:** 2014-10-13

**Authors:** Katherine R. Smith, Laureen Barthman-Thompson, William R. Gould, Karen E. Mabry

**Affiliations:** 1 Department of Biology, New Mexico State University, Las Cruces, New Mexico, United States of America; 2 California Department of Fish and Wildlife, Stockton, California, United States of America; 3 Department of Economics, Applied Statistics and International Business, New Mexico State University, Las Cruces, New Mexico, United States of America; Institut Pluridisciplinaire Hubert Curien, France

## Abstract

The northern salt marsh harvest mouse (*Reithrodontomys raviventris halicoetes*) is an endangered species endemic to the San Francisco Bay Estuary. Using a conservation behavior perspective, we examined how salt marsh harvest mice cope with both natural (daily tidal fluctuations) and anthropogenic (modification of tidal regime) changes in natural tidal wetlands and human-created diked wetlands, and investigated the role of behavioral flexibility in utilizing a human-created environment in the Suisun Marsh. We used radio telemetry to determine refuge use at high tide, space use, and movement rates to investigate possible differences in movement behavior in tidal versus diked wetlands. We found that the vast majority of the time salt marsh harvest mice remain in vegetation above the water during high tides. We also found no difference in space used by mice during high tide as compared to before or after high tide in either tidal or diked wetlands. We found no detectable difference in diurnal or nocturnal movement rates in tidal wetlands. However, we did find that diurnal movement rates for mice in diked wetlands were lower than nocturnal movement rates, especially during the new moon. This change in movement behavior in a relatively novel human-created habitat indicates that behavioral flexibility may facilitate the use of human-created environments by salt marsh harvest mice.

## Introduction

Animal behavior has the potential to contribute substantially to conservation efforts, but the field of conservation behavior has been slow to develop [1]–[6]. Only recently have attempts been made to develop unifying principles and overarching frameworks for the study of conservation behavior [7]–[9]. Because behavior is one of the most flexible traits an animal may possess, it represents a promising mechanism by which species may cope with human-induced rapid environmental change (HIREC) [10]–[13].

Behavioral flexibility is the ability of an animal to modify its behavior under different environmental conditions [10], [14], [15]. Behavioral responses are often rapid and reversible, and afford animals some degree of control over their external stimuli by choosing their surrounding environment [12]. Appropriate behavioral responses to environmental change are vital for animals that live in naturally heterogeneous environments, and will likely be critical for the persistence of many species in the face of HIREC [12].

Both natural environmental heterogeneity and the effects of HIREC occur across time and space, and this environmental variability affects species occurrence, distribution, abundance, population stability, and individual behavior [16]–[18]. Some habitat types are relatively stable, while others are highly variable in their natural state. For example, wetland biota is influenced by a variety of both predictable (tides, seasonal flows) and stochastic (flood, drought) changes on a variety of time scales [19]. Anthropogenic changes to wetlands, such as modification of tidal inundation regimes through the construction of earthen dikes/levees, can greatly interfere with these natural patterns of heterogeneity. Because coastal wetlands are both highly variable in their natural state and have been heavily modified by human activities, organisms that live in coastal wetlands present opportunities for the study of both behavioral flexibility in response to natural heterogeneity and behavioral responses to HIREC.

We investigated the response of the endangered northern salt marsh harvest mouse (*Reithrodontomys raviventris halicoetes*) to both natural and anthropogenic environmental heterogeneity. The salt marsh harvest mouse is an ideal species in which to study behavioral flexibility and responses to HIREC, because it is endemic to changeable wetland habitats and currently occurs in both natural tidal (full tidal influence) and anthropogenically-altered (diked) wetlands.

Features of salt marsh harvest mouse biology that are consistent with adaptation to tidal environments become clear when comparisons are made to the sympatric congener the Western harvest mouse (*R. megalotis*), which occurs in tidal marshes but also in a variety of other habitat types. Compared to Western harvest mice, the salt marsh harvest mouse is a stronger swimmer and is more capable of consuming sea water [20]. They are also capable of entering torpor [20], a mechanism thought to aid small mammals in coping with osmotic stress [21], and are more active during warmer daytime temperatures which allows for a lower resting metabolic rate than activity during cool, damp nights [20], [22]. Finally, as a species that naturally resides in a highly changeable environment, the salt marsh harvest mouse may possess a high level of behavioral flexibility that predisposes it to alter its behavior to utilize resources in novel habitat types, such as diked wetlands [23].

Understanding how salt marsh harvest mice use their habitat during daily tidal flooding is especially important because tidal restoration is the primary conservation measure for preserving this species which is endangered due to dramatic habitat loss (∼90%) and faces further loss due to predicted sea level rise [24]–[27]. As the only terrestrial mammals that is entirely restricted to coastal marshes, the salt marsh harvest mouse must somehow avoid drowning during high tides [28]. It has long been believed that salt marsh harvest mice spend the majority of their time in wetlands, and move upland only to escape tide and flood waters [20].

Since the 1950s, researchers have attempted to characterize salt marsh harvest mouse behavior during tidal inundation to determine whether mice move vertically into tall vegetation or horizontally upland to escape the tide [20], [29]–[31]. In tidal wetlands salt marsh harvest mice typically experience tidal flooding twice a day. In contrast, diked wetlands are flooded continuously during the rainy season (approximately October through March), and dried out for the remainder of the year. Thus, the patchwork of modified and unmodified habitat in the San Francisco Bay Estuary presents the opportunity to study habitat use of mice in both highly changeable natural (tidal) habitat and highly modified (diked, surrounded by levees with water control structures) habitat that is temporally more stable.

Conclusions from previous studies of the behavior of the salt marsh harvest mouse at high tide have been split on the question of how mice deal with daily tidal inundation of their habitat. Some concluded based on trapping data or visual observations that animals move out of tidal wetlands and into upland areas or onto levees to escape rising waters [29], [30], and others concluded that they remain in tall, dense vegetation over water where they can easily move about in the thatch layer [20], [31]. However, although trapping methods allow researchers to determine that a mouse had been at a location at some point during the night, it is impossible to tell from these previous trapping studies where mice were during the time that the habitat was actually flooded. The contrasting conclusions of previous studies suggest that the only way to definitively understand salt marsh harvest mouse habitat use during high tide is through the use of radio telemetry [31].

The objective of this study was to determine how salt marsh harvest mice respond to both natural environmental heterogeneity and HIREC, focusing on the effects of regular tidal inundation. We approached this investigation from a conservation behavior perspective which uses general principals of animal behavior to address conservation issues [32]. We applied two questions from a recently developed conservation behavior framework [7]. One focus of the Berger-Tal et al. framework stresses performing behavior-based management, which considers behavior in conservation decision making protocols [7]. In our case, answering the question *Do salt marsh harvest mice move vertically (into tall vegetation) or horizontally (into upland areas) to escape the high tide*? will allow managers develop restoration and enhancement priorities in the little habitat that remains, and is a research priority explicitly identified in the recovery plan for this species [26]. A second focus of the framework addresses if and how HIREC affects behavior, in the case of this endangered species, *Do mice in anthropogenically altered diked wetlands behave differently than those subject to tidal influence*? [7].

To answer these questions, we tracked mouse movements during high tide events in natural tidal wetlands using radio telemetry to identify the type of refuge used to escape incoming water. During the same periods, we tracked mice in adjacent diked wetlands. This allowed us to determine space use and estimate movement rates in response to high tides, as well as independent of tidal influence in diked habitats. With regard to our first question, we predicted that during tidal flooding, mice would remain in tall vegetation over water. Our predictions for the second question depended on the results of the first. If the prediction that mice remain in tall vegetation during the high tide was confirmed, we expected that movement distances (and thus rates) in natural tidal and diked wetlands would be similar. In contrast, if mice instead moved long distances upland to escape the tide, then we expected that movement rates in natural tidal wetlands would be greater than those in diked wetlands.

## Methods

### Ethical note

The research reported here was conducted under an approved IACUC protocol from New Mexico State University (2011–013) and a Memorandum of Understanding between the California Department of Fish and Wildlife and the United States Fish and Wildlife Service for handing of endangered species, with direct involvement and supervision by the California Department of Fish and Wildlife. All procedures were consistent with the guidelines for the use of wild mammals in research from both ASAB/ABS and the American Society of Mammalogists.

### Study Area

**Figure 1:**
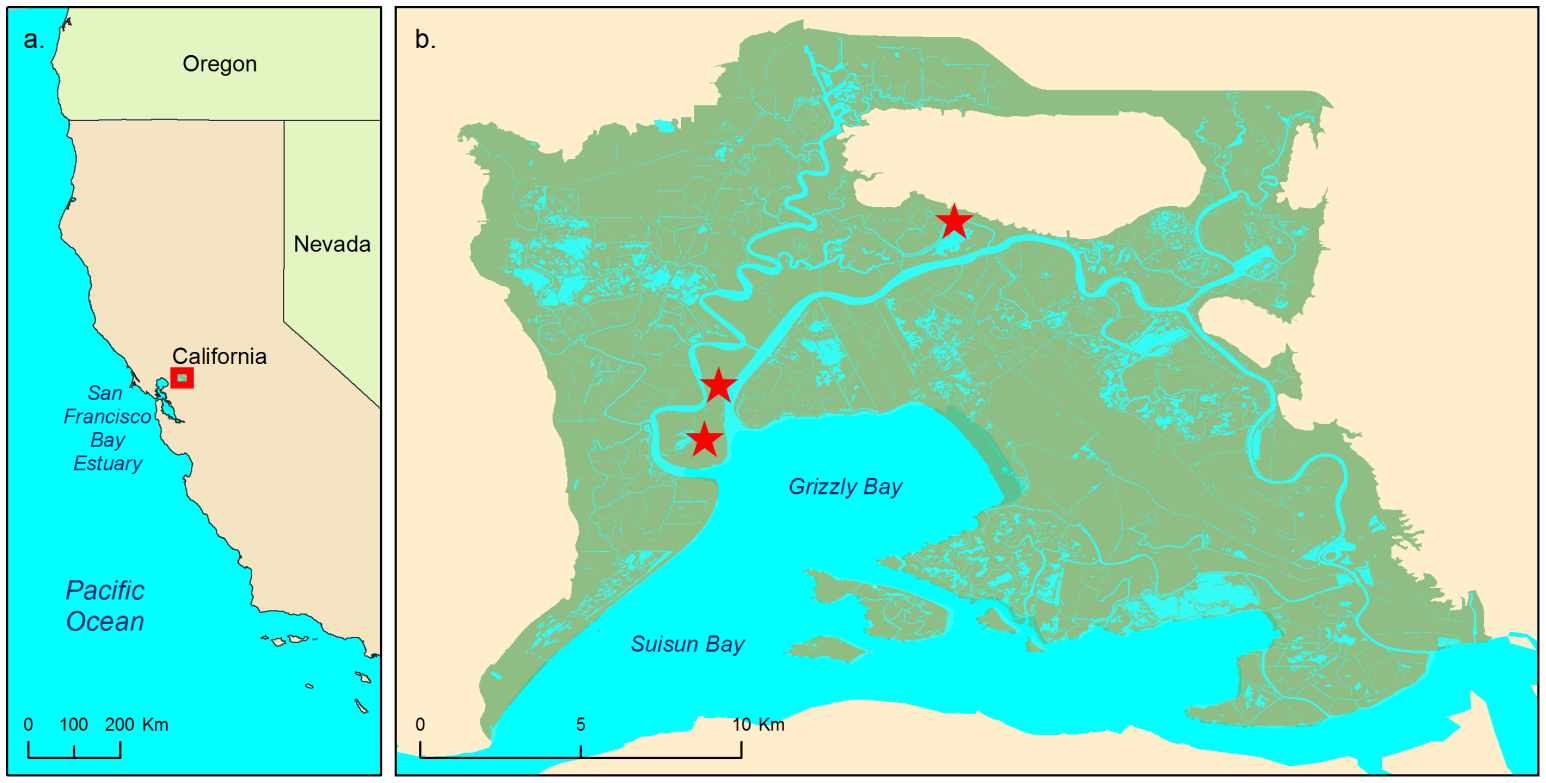
Maps of study location and areas. (a) The south-west coast of the United States with the location of the Suisun Marsh indicated by the boxed area. (b) The Suisun Marsh with 3 study blocks indicated by red stars. Data Source: California Department of Fish And Game: Vegetation - Suisun Marsh 2009 [ds711], Ocean Delta Water; California Digital Conservation Atlas. © Katherine Smith

We conducted this study between May and August 2011 in the Suisun Marsh in Solano County, CA, USA (38° 11′ 15.86″ N, 122° 3′ 52.67″ W), which is a large wetland complex in the San Francisco Bay Estuary (Fig. 1a). We established six study areas which were grouped into three blocks, each containing one natural tidal and one diked wetland. Study areas were established on public lands with the permission of the California Department of Fish and Wildlife and the Suisun Resource Conservation District. Paired wetlands (one tidal and one diked) within each block were 100 to 600 m apart and separated by levee roads or sloughs, and the three blocks were 1 100 to 8 500 m apart (Fig. 1b). Although salt marsh harvest mice are capable of moving distances similar to the distances between our study wetlands, we did not observe movement between these wetlands during our study. All tidal wetlands were subject to full natural tidal action. All diked wetlands were cut off from water access for the summer and were essentially dry.

Vegetation on the study sites included pickleweed (*Salicornia virginica*, also known as Virginia glasswort), Olney's threesquare bulrush (*Schoenoplectus americanus*, also known as chairmaker's bulrush), cattail (*Typha* spp.), saltgrass (*Distichlis spicata*), tule (*Sc. acutus*), common reed (*Phragmites australis*), Baltic rush (*Juncus balticus*) and other halophytic species. Plant community composition was broadly similar between natural tidal and diked areas, although tidal areas tended to be dominated by reeds and rush (mean height ±1 *SE*  = 138.13±27.84 cm), while the diked areas tended to be dominated by pickleweed (mean height ±1 *SE*  = 42.40±6.00 cm). Although the vegetation was similar overall, diked wetlands had a higher proportion of bare ground, which lead to a lower calculated mean height.

### Live Trapping

During more than 3 500 trap nights, we captured small mammals using collapsible Sherman live traps (SFA, 7.62×8.89×23.50 cm; H.B. Sherman Traps Inc., Tallahassee, FL). We placed trap grids in vegetation types known to support large salt marsh harvest mouse populations, including pickleweed and Olney's threesquare bulrush [33]–[35]. Traps were placed at 10 meter spacing within grids consisting of 50 or 100 traps, depending upon wetland configuration and mouse density. We placed traps flat on the ground in diked wetlands. In natural tidal wetlands, we placed traps above the water line, nested securely in the vegetation. We placed paperclips at the top of trap doors to leave a small space between the door and the body of the trap to prevent the tails of salt marsh harvest mice from getting caught when traps closed. We baited traps with ground walnut and mixed birdseed, and added cotton batting to the traps for warmth. Traps were opened at sunset, and checked and closed at sunrise. We trapped and tracked mice simultaneously in paired natural tidal and diked wetlands within each block during the same moon phases. At each block, trapping was initiated 4–5 days before the highest tide of the full and new moons. Tracking began 2–3 days before the highest high tide of the lunar phase, and typically continued until 2–3 days after the highest tide.

### Radio Tracking

To maximize the potential effect of tidal inundation on movement, we tracked mice during several days surrounding the full moon, which is typically when the highest tides occur. To control for potential effects of bright lunar illumination during the full moon, we also tracked during the new moon when tides are also very high but lunar illumination is low. We tracked mice during sequential full and new moon phases for each block, with a different set of mice tracked during each lunar phase. This yielded a 2×2 factorial arrangement with four “treatments”: tidal wetland during the full moon, tidal wetland during the new moon, diked wetland during the full moon, and diked wetland during the new moon.

To track movements of salt marsh harvest mice, we fitted individuals with BD-2NC radiotransmitters (Holohil, Inc., Carp, Ontario, Canada). Transmitters were equipped with the smallest battery possible, resulting in a total transmitter package mass of approximately 0.5 g. Because of the small size of salt marsh harvest mice (adult mass  = 7.6–14.5 g, [20]), only the largest individuals could be collared in order to meet the recommendation that collars not exceed 5% of body mass [36]. Because females of sufficient size were commonly pregnant or lactating, we used only males weighing ≥10 g. We collared up to five of the largest individuals per wetland per lunar cycle (up to ten individuals per block per lunar cycle), yielding a total of 42 mice, 23 in the tidal wetlands and 19 in the diked wetlands. We collared mice in the field without using anesthesia and held them for observation for ten minutes following the collaring procedure; none showed any adverse reactions. Following the observation period, we released mice at their capture location and began tracking their movement during the second high tide following release (approximately 12 hours later). We tracked each individual for up to six days (mean; *SD*  = 4.3; 0.99 days, min  = 2 days, max  = 6 days), collecting location data approximately once an hour for 2 to 3 hours on either side of the peak of both diurnal and nocturnal high tides. When tracking was complete, we re-trapped mice and removed their collars. Due to some dropped collars and unexpectedly short battery lives, not all collared individuals yielded sufficient data for analysis.

We used standard radiotracking methods to estimate individual locations, using triangulation from known coordinates [37], with two bearings recorded per location by two observers (a total of four bearings from different points were used to triangulate each location). By allowing the location of animals from a distance, triangulation minimized the influence of observer presence on mouse movement and reduced damage to habitat. It also allowed us to track mice in flooded areas that were inaccessible to humans on foot. Location estimates were generated using Lenth's technique for maximum-likelihood estimates (MLE) in the computer program LOCATE III [38]. To ensure that mice retained their collars and were not caught in vegetation, we homed in on each mouse following each high tide. We then recorded location coordinates using a handheld GPS unit with submeter accuracy (Trimble GeoExplorer 3; Trimble Navigation Limited, Sunnyvale, CA, USA). Homing locations also allowed us to estimate the accuracy of triangulated locations. When a homing location was recorded within 15 minutes of a triangulated location, we calculated the distance between the homing location and the triangulated location to estimate triangulation error. The distance between triangulated and homed points was 15.51; 8.83 meters (mean; *SD*, N = 37). However, this error distance is probably an overestimate, because mice are capable of moving large distances in short periods of time, and because animals often move away from researchers during the homing process, which would increase the distance between triangulated and homed locations.

### Determining Refuge Use

Our first goal was to determine whether salt marsh harvest mice moved vertically into emergent vegetation or horizontally into upland areas during the high tide. We examined mouse locations that were recorded within one hour of the high tide in relation to the tide height. We created maps of inundation for each tidal site using water surface data. To measure water surface level, we placed tide level markers throughout tidal areas within and around trap grids at 20 meter spacing, and measured the maximum height reached by water following each high tide. Tide markers were wooden dowels marked each centimeter and dusted with colored chalk. Tide height was estimated by examining dowels following high tide and noting the height to which chalk had been washed away. We recorded coordinates of all tide markers using a handheld GPS unit with submeter accuracy (Trimble GeoExplorer 3; Trimble Navigation Limited, Sunnyvale, CA, USA).

We input all tide measurements as points into ArcMap 10 and interpolated a raster surface using an Inverse Distance Weight method (Inverse Distance Weight, ArcGIS 10.0; Environmental Systems Research Institute, Redlands, CA, USA) which overlaid well with aerial imagery and was consistent with observations in the field. Over this raster surface, we overlaid all mouse locations recorded “during” the high tide. Points occurring within one hour before or after the time of the highest point of the tide were categorized as “during” the high tide. If a mouse location fell in an area where water level was ≤1 cm we considered it “upland”. If a mouse location fell in an area where water level was >1 cm it was considered “over water”. If a location was determined to be upland, we assumed the mouse moved horizontally to escape the tide. If a location was categorized as over water, we assumed that the mouse had remained in emergent vegetation to escape the tide. Refuge use was determined using the lowest high tide of both the full and new moon periods during which mice were tracked, thus represented the most conservative categorization of locations as over water. That is, locations were only considered to be over water if they would have been over water during the lowest high tide that we observed at a site. Locations that fell within 15 m of the boundary between inundated and unindated areas were excluded from analysis of refuge use. Because these “boundary” locations were within the average triangulation error distance, we could not definitively determine whether they were over water or not.

We also performed an analysis using minimum convex polygons (MCP) of salt marsh harvest mouse locations to test for differences in space use between mice in tidal and diked wetlands. Individual locations were grouped as “before”, occurring more than one hour before high tide; “during”, within one hour before or after the high tide; and “after”, occurring more than one hour following the high tide. Using the Minimum Bounding Geometry-Convex Hull tool in ArcMap we created “before”, “during”, and “after” polygons for each mouse during each time period for which we recorded ≥6 locations. Then using the Intersect tool in ArcMap we calculated what percentage of the “before” and “after” MCPs overlapped the “during” MCP.

### Estimating Movement Rates

Our second goal was to determine whether there were differences in movement distances between mice in natural tidal wetlands and mice in diked wetlands. However, because the time intervals between location estimates were not equal, it was necessary to first standardize movement data as rates. Movement rate is the Euclidean distance between two consecutive point locations divided by the time elapsed between those points. We calculated movement rates using all locations of an individual that were recorded less than three hours apart before, during and after high tide events (mean ±1 *SE*  = 83.94±1.40 minutes between successive points, min  = 21 minutes, max  = 180 minutes). Based on average times of sunrise and sunset during the study, we categorized movements occurring between 0601 and 2000 hours as “diurnal” and movements between 2001 and 0600 as “nocturnal”, and calculated average movement rates separately for day and night for each mouse. We omitted from analysis 18 movement rates that spanned the diurnal/nocturnal time cut-offs. The number of movement rates per mouse per time of day (diurnal vs. nocturnal) ranged from 1 to 17, with an average of 7.28; 3.87 (mean; *SD*) rates per individual per time of day (total movement rates  = 411). Mice with <5 movement rates were excluded from analysis, leaving a total of 29 mice for which we analyzed movement rate data, 15 in tidal wetlands and 14 in diked wetlands.

### Analysis

Statistical analysis of the MCP data was performed using paired t-tests to compare space use before and after the high tide to space use during the high tide. Statistical analysis of movement rates was conducted using PROC MIXED in SAS 9.3 (SAS Institute Inc., Cary, NC, USA). We used a replicated block design with blocks and the combination of wetland type and moon phase as fixed effects (four “treatments”: tidal full, tidal new, diked full, and diked new), and mouse identification number as a nested random effect, with time of day (diurnal or nocturnal) as a repeated factor. Tukey post-hoc tests were used to detect differences among treatments using an alpha level of 0.05 for statistical significance. Only locations from mice in natural tidal wetlands were used for the refuge use analysis, while locations from all mice with sufficient data were used in the space use and movement rate analysis.

## Results

### Refuge Use

**Figure 2:**
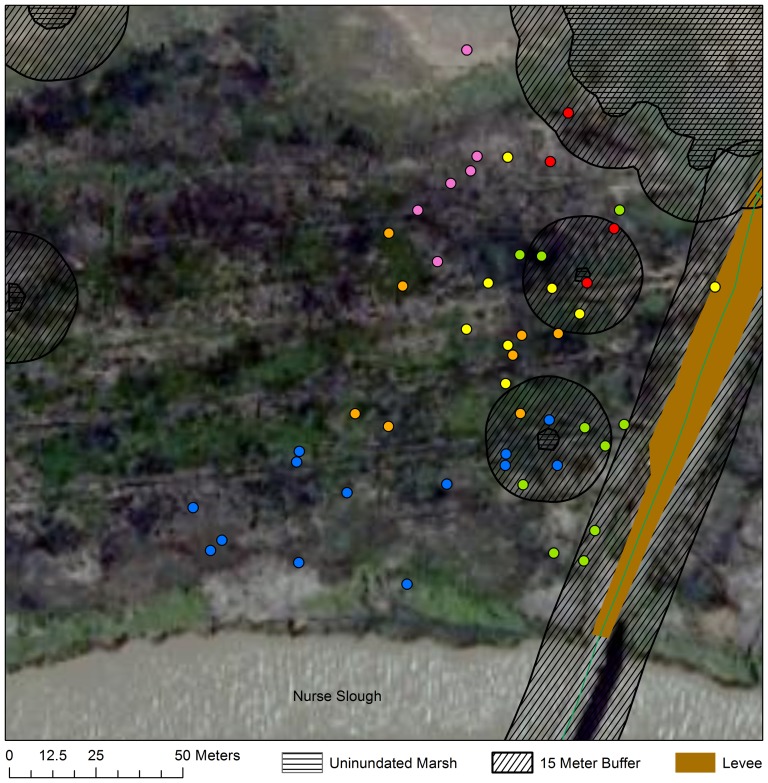
Individual locations at Joice Island Tidal. Individual salt marsh harvest mouse locations at high tide at Joice Island Tidal. Each individual is represented by a different color. Each point represents a mouse location within one hour of the high tide. Data Source: California Department of Fish And Game: NAIP 2010 Aerial Imagery. © Katherine Smith

**Figure 3:**
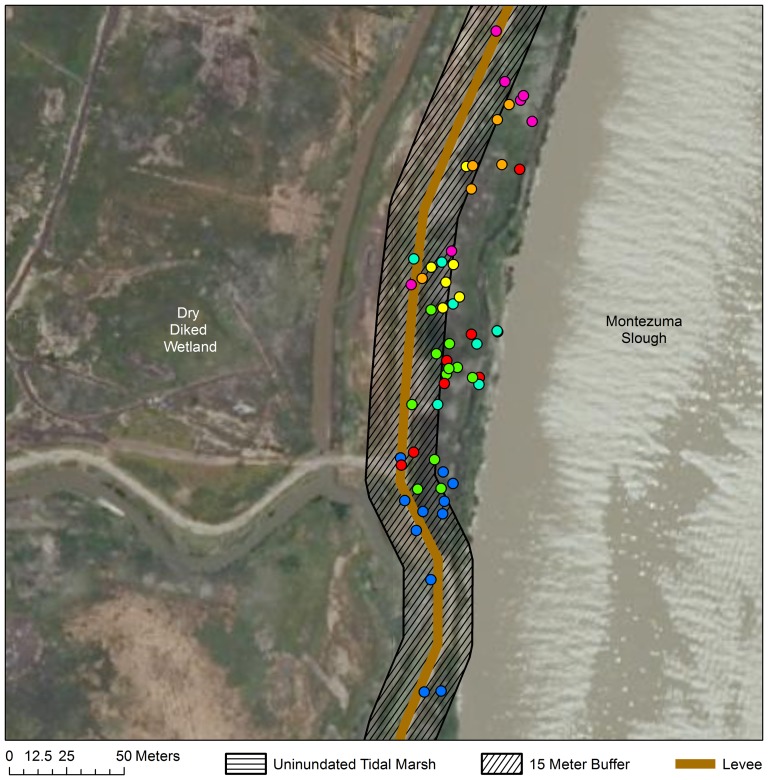
Individual locations at Lower Joice Island North Tidal. Individual salt marsh harvest mouse locations at high tide at Lower Joice Island North Tidal. Each individual is represented by a different color. Each point represents a mouse location within one hour of the high tide. Data Source: California Department of Fish And Game: NAIP 2010 Aerial Imagery. © Katherine Smith

**Figure 4:**
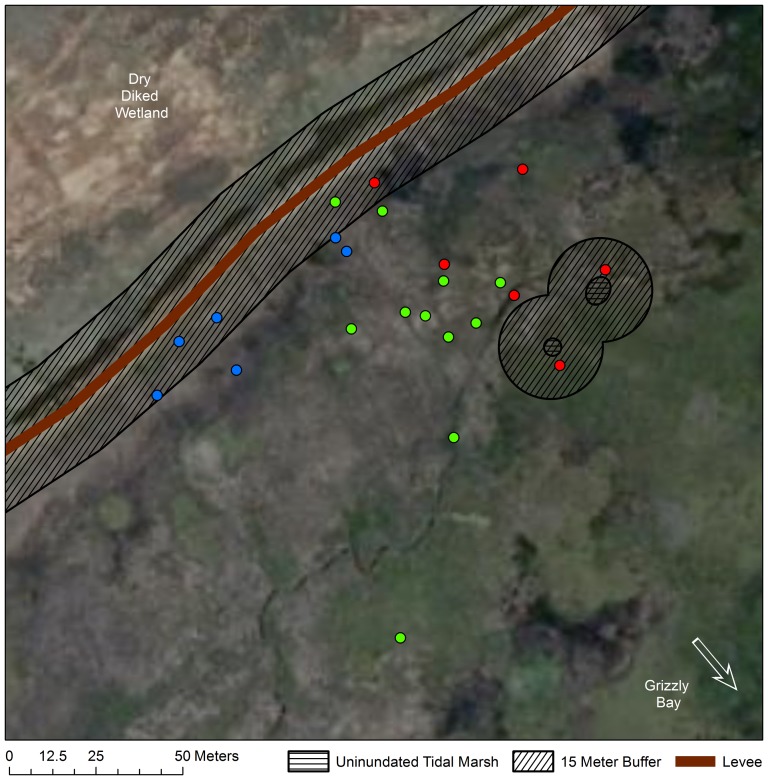
Individual locations at Lower Joice Island South Tidal. Individual salt marsh harvest mouse locations at high tide at Lower Joice Island South Tidal. Each individual is represented by a different color. Each point represents a mouse location within one hour of the high tide. Data Source: California Department of Fish And Game: NAIP 2010 Aerial Imagery. © Katherine Smith

For mice in natural tidal wetlands, we recorded a total of 167 locations within 1 hour of high tide from 16 mice (mean; *SD*  = 11.48; 4.02 points per mouse). Forty-two of these 167 locations (∼25%) were within 15 m of the boundary between areas that we considered “upland” versus “over water” (Figs. 2–4). Because these locations were within our average triangulation error from the boundary, we could not classify them to either category with confidence, and they were eliminated from further analysis. Of the remaining 126 locations, 125 (>99%) were definitively in vegetation over water and 1 (<1%) was upland. It is worth noting that even if locations falling within 15 m of the boundary between inundated and unindated areas were included, only 6% (10/167) would have been categorized as “upland”. Because we used the most conservative definition of “over water” (e.g., that the position would have been over water during the lowest high tide during which mice were tracked), 6% is likely an overestimate of the frequency with which mice used upland refuges.

**Figure 5:**
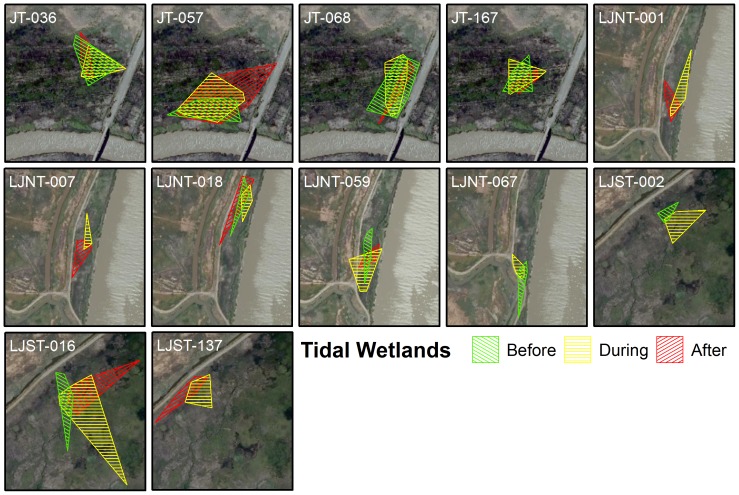
Minimum convex polygons for mice in tidal wetlands. Minimum convex polygons for salt marsh harvest mice in tidal wetlands. Green polygons represent points occurring more than one hour before high tide. Yellow polygons represent points falling within one hour before or after high tide. Red polygons represent points occurring more than one hour following high tide. Each block represents an individual mouse. Some blocks have all three polygons, while those that lack 6 or more individual points during the before or after time period, only have two polygons.

**Figure 6:**
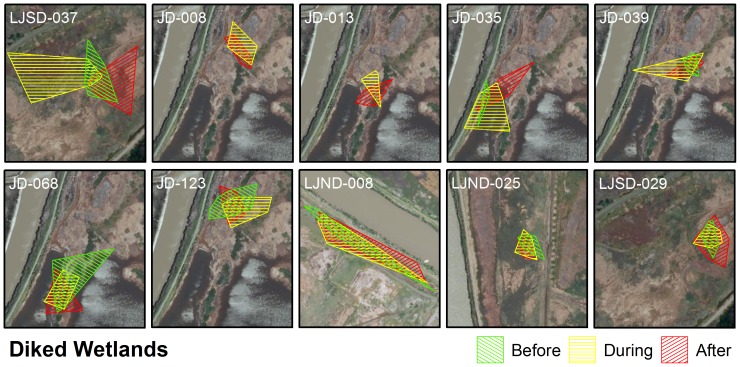
Minimum convex polygons for salt marsh harvest mice in diked wetlands. Green polygons represent points occurring more than one hour before high tide. Yellow polygons represent points falling within one hour before or after high tide. Red polygons represent points occurring more than one hour following high tide. Each block represents an individual mouse. Some blocks have all three polygons, while those that lack 6 or more individual points during the before or after time period, only have two polygons.

When comparing the MCP of points for individuals before (t-test: t_15_ = 1.327, P = 0.20; diked: n = 8, mean ± SE  = 0.61±0.09; tidal: n = 9, mean ± SE  = 0.44±0.09) and after (t-test: t_18_ = −0.55, P = 0.59; diked: n = 10, mean ± SE  = 0.56±0.09; tidal: n = 10, mean ± SE  = 0.49±0.08) high tide to the MCP during high tides we saw no significant difference between tidal and diked wetlands (Figs. 5–6). We found no evidence that salt marsh harvest mice in tidal wetlands shift their space use more or less than mice in diked wetlands during the same high tide periods.

### Movement rates

**Figure 7:**
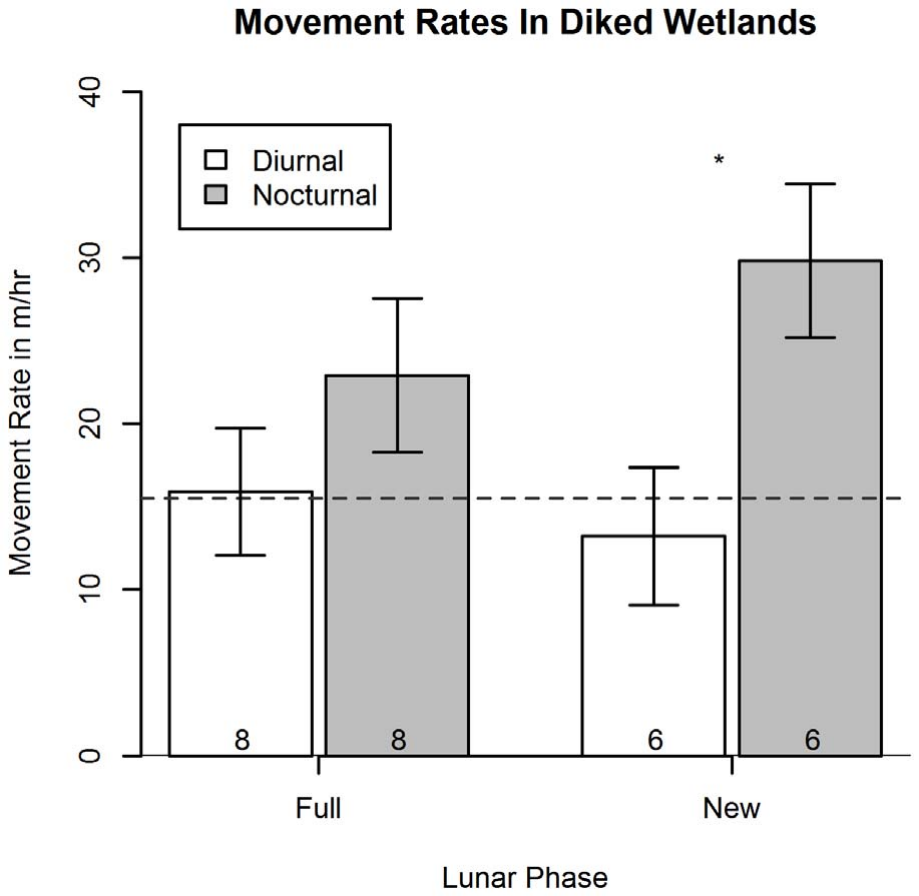
Movement rates for mice in diked wetlands. Movement rates for salt marsh harvest mice in diked wetlands. Dashed line represents 15 meters, corresponding to the level of error associated with the inundated/uninundated area boundary. Significant difference is marked with an asterisk; *P* = 0.012.

**Figure 8:**
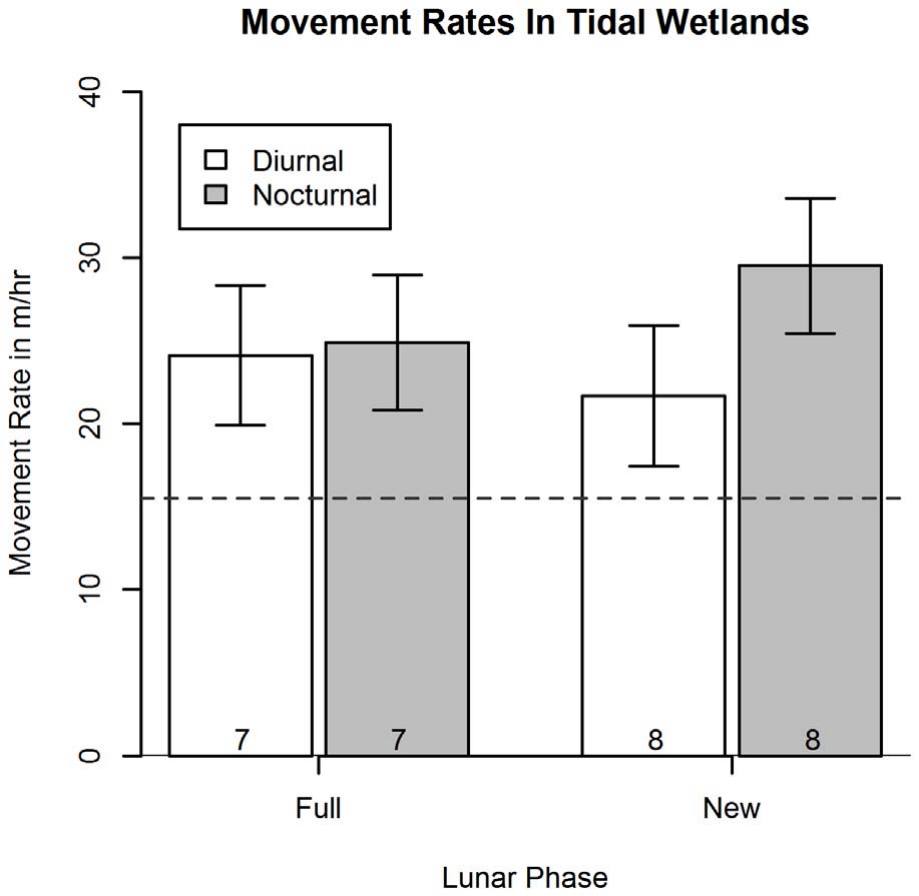
Movement rates for mice in tidal wetlands. Movement rates for salt marsh harvest mice in tidal wetlands. Dashed line represents 15 meters, corresponding to the level of error associated with the inundated/uninundated area boundary.

Because mice remained over water instead of moving large distances to use upland refuges to escape high tides in natural tidal wetlands, it was not surprising that we found no significant difference in movement rates among treatments (combinations of wetland type and moon phase; [repeated-measures ANOVA: F_3,24_ = 0.92, P = 0.44]). As expected for a primarily nocturnal species, we did find an effect of time: diurnal movement rates were lower than nocturnal movement rates (repeated-measures ANOVA: F_1, 23_ = 7.46, P = 0.01). Post-hoc comparisons of diurnal and nocturnal movement rates within treatments suggested that this difference was primarily due to diked habitats, in which diurnal movement rates during the new moon were considerably lower than nocturnal movement rates (Tukey post-hoc test: P = 0.012, Fig. 7). In contrast, in natural tidal wetlands, diurnal and nocturnal movement rates were very similar (Table 1). These data indicate that in natural tidal wetlands, mice are moving almost as much during the day as at night (Fig. 8).

**Table 1:** Average movement rates of salt marsh harvest in meters per hour for treatments.

		Nocturnal		Diurnal	
Wetland Type	Moon Phase	Mean	*SE*	Mean	*SE*
Tidal	Full	24.89	4.22	24.11	4.22
Tidal	New	29.51	4.08	21.67	4.07
Diked	Full	22.91	4.14	15.91	3.82
Diked	New	29.82	4.63	13.23	4.63

All rates before, during, and after high tides are included in this analysis.

## Discussion

Understanding how animals cope with both naturally-occurring heterogeneity and HIREC in their environment can be crucial to the conservation of threatened species such as the salt marsh harvest mouse [11], [39]. Behavioral flexibility represents a coping mechanism likely to be crucial for many animals in the face of anthropogenic change in their environment [11]–[13]. The purpose of this study was to determine how salt marsh harvest mice respond to both natural heterogeneity and HIREC. Following the framework of Berger-Tal et al. [7], we answered two questions. First, we considered behavior-based management and asked *do salt marsh harvest mice move vertically (into tall vegetation) or horizontally (into upland areas) to escape the high tide*? Determining high tide refuge use by the salt marsh harvest mouse is a high conservation priority for this endangered species [26]. We used radio telemetry to show that the vast majority of the time, male salt marsh harvest mice in the Suisun Marsh used emergent vegetation to escape tidal inundation, suggesting that maintenance of healthy intertidal vegetation is important in the conservation of small mammals and protection of biodiversity in this system. This conclusion is supported by extensive live-trapping data from a previous study that indicated that salt marsh harvest mice are only rarely trapped in upland areas [35], and is not surprising in the Suisun Marsh where vegetation is generally tall and thick providing extensive structure and cover. Our behavioral results will allow for improved habitat management, allowing managers to concentrate conservation efforts on maintaining intertidal habitat rather than expending efforts enhancing upland habitats that salt marsh harvest mice rarely use.

Secondly, we addressed whether and how HIREC causes changes in behavior [7]; specifically, do salt marsh harvest mice in anthropogenically altered diked wetlands behave differently than those subject to tidal influence? The lack of a significant difference in nocturnal movement rates between natural tidal and diked wetlands would suggest that this anthropogenic change has not affected the behavior of this species. However, diurnal movement rates were consistently lower than nocturnal movement rates in diked wetlands, a pattern not observed in tidal wetlands (Figs. 7–8). This result indicates that mice in human-influenced diked wetlands may have altered their diurnal movement behaviors in anthropogenically-modified diked wetlands, moving less during the day. Current telemetry efforts support these results and are revealing similar trends throughout the day, not just during high tides (K.R. Smith, unpublished data).

There are two potential explanations for the observed decrease in movement rate in diked wetlands during the day. First, anthropogenic habitat modification may have had an adverse effect on salt marsh harvest mice. For example, differences in habitat structure or a lack of inundation may either allow for increased access by diurnal predators or decreased cover for mice, potentially leading to reduced diurnal movement rates in diked wetlands (mice are *forced* to seek refuge during the day). A second potential explanation is that removal of tidal influence has instead had a positive effect on salt marsh harvest mice. For example, mice in diked wetlands may have increased availability and/or accessibility to resources, or an increased ability to cache resources, which could allow mice to restrict their foraging to night-time hours, remaining in refuges during the day when predation pressure is high (mice are *choosing* to seek refuge during the day) [40]. This possibility is supported by our observations of salt marsh harvest mice using underground refuges, and taking cover in voids below a hummocks of cattails during the day in three separate diked wetlands, behavior that would not be possible in a tidal wetland [41].

Habitat loss has been the primary threat to the salt marsh harvest mouse, and with the level of existing and continuing development in the San Francisco Bay Estuary [42]–[44], subsidence, and sea level rise [28], [45] the marshes utilized by the salt marsh harvest mouse are vulnerable to additional habitat loss. Every remaining hectare of salt marsh harvest mouse habitat is crucial, and maximizing the value of this habitat through restoration and enhancement will be key to the persistence and recovery of the species [26], [45]. There are currently at least three plans that call for restoration of diked wetlands to tidal influence in the Suisun Marsh in the immediate future: Suisun Marsh Habitat Management, Preservation, and Restoration Plan [46], the Bay Delta Conservation Plan [47], and the Fish Restoration Program Agreement [48]. Refuge use by salt marsh harvest mice is a behavior that must be taken into account when planning and implementing these tidal restorations.

There is currently little research investigating the differences in value of various habitat types to the salt marsh harvest mouse. Our trapping was not designed to assess effects of wetland type on populations or densities of salt marsh harvest mice, but another study suggests that diked wetlands may be at least as valuable to conservation efforts as tidal wetlands, by supporting higher densities of mice [35]. Additionally, large populations of salt marsh harvest mice have recently been found in diked wetlands, even during flooded winter months (K.R. Smith, unpublished data). This suggests that this species is capable of thriving in flooded diked wetlands by remaining in tall vegetation over standing water. Given that much of the remaining salt marsh harvest mouse habitat in the Suisun Marsh exists as diked wetlands [45], the behavior and ecology of individuals living in these modified habitats warrants further exploration.

It is important to note that our findings may not necessarily apply to the entire range of the salt marsh harvest mouse. In the Suisun Marsh tidal vegetation is tall and marshes are wide, and there is an abundance of diked (21 044 hectares) and upland (11 210 hectares) habitat available, while only 21% of historic tidal habitat remains (2 550 hectares) [49]. The two other major strongholds for this species differ in marsh width and vegetation type: in San Pablo Bay many marshes are wide, but vegetation is short, and in the South San Francisco Bay marshes are narrow and vegetation is short [49]. In these areas, mice may employ alternate strategies when escaping the tide; for example, if vegetation is shorter than the water height at high tide, mice may have no choice but to move upland where they may be exposed to predation.

Finally, due to a number of limiting factors including minimum transmitter size and logistical constraints, we were unable to track female or young salt marsh harvest mice, so any speculation about their behavior should be made with care. Indeed, a recent study of another endangered wetland rodent, the New Mexico jumping mouse (*Zapus hudsonius luteus*), found different patterns of habitat use by males and females [50]. As technology advances and radio collars become smaller, it would be prudent to repeat this study using both male and female mice, as well as sub-adults and juveniles.

The results of this study provide insight to a question that researchers have been investigating for over 55 years: where do salt marsh harvest mice go during the high tide [29]? This study increases our understanding of the behavior of endangered salt marsh harvest mice in tidal and diked wetlands, suggesting that these small mammals may be capable of coping with the challenge of living in tidal wetlands with little to no emergent land as long as vegetation is sufficiently thick and tall. They also indicate that small mammals may exhibit different behaviors in diked wetlands than in tidal wetlands, highlighting the need to consider behavior when planning for conservation or management projects and shedding light on the need for comprehensive management strategies that account for potential behavioral differences in populations. Behavioral flexibility may be critical for threatened species in the face of HIREC [12]. As we cause further changes to the environment both directly, through processes such as urbanization, and indirectly, though processes such as climate change, the work of behaviorists will be critical in understanding behavioral responses of threatened species, like the salt marsh harvest mouse, to HIREC [6], [11], [51].
